# Effects of cold-water immersion on health and wellbeing: A systematic review and meta-analysis

**DOI:** 10.1371/journal.pone.0317615

**Published:** 2025-01-29

**Authors:** Tara Cain, Jacinta Brinsley, Hunter Bennett, Max Nelson, Carol Maher, Ben Singh

**Affiliations:** Alliance for Research in Exercise Nutrition and Activity (ARENA), Allied Health and Human Performance, University of South Australia, Adelaide, Australia; Hamasaki Clinic, JAPAN

## Abstract

**Background:**

Cold-water immersion (CWI) has gained popularity as a health and wellbeing intervention among the general population.

**Objective:**

This systematic review and meta-analysis aimed to evaluate the psychological, cognitive, and physiological effects of CWI in healthy adults.

**Methods:**

Electronic databases were searched for randomized trials involving healthy adults aged ≥ 18 years undergoing acute or long-term CWI exposure via cold shower, ice bath, or plunge with water temperature ≤15°C for at least 30 seconds. Outcomes of interest were sleep, stress, fatigue, energy, skin health, immunity, inflammation, mental wellbeing, depression, anxiety, mood, concentration, and alertness or focus. Meta-analyses were conducted using RevMan software (version 5.4), applying random effects models to calculate standardized mean differences (SMD) between pre- and post-CWI exposure outcomes. Risk of bias was assessed using the PEDro scale.

**Results:**

Eleven studies were included, comprising 3177 total participants and a mean PEDro score of 6.4 (n = 7 moderate quality, n = 4 high quality). CWI interventions were performed in baths (n = 10) or showers (n = 1) at temperatures ranging from 7°C to 15°C and durations ranging from 30 seconds to 2 hours. The meta-analysis revealed significant increases in inflammation immediately (SMD: 1.03, [95% CI: 0.37, 1.68], p <  0.01) and 1 hour post CWI (SMD: 1.26, [95% CI: 0.59, 1.94], p <  0.01), indicating an acute inflammatory response. A significant reduction in stress was observed 12 hours post-CWI (SMD: –1.00, [95% CI: –1.40, –0.61], p <  0.01), however, no significant effects on stress were detected immediately (SMD: –0.09 [95% CI: –0.45, 0.63], p >  0.05), 1 hour (SMD: –0.29 [95% CI: –0.66, 0.08], p >  0.05), 24 hours (SMD: –0.06 [95% CI: –0.50, 0.38], p >  0.05), or 48 hours (SMD: 0.09 [95% CI: –0.28, 0.46], p >  0.05) post-exposure. While meta-analysis showed no significant effects on immune function immediately (SMD: –0.16 [95% CI: –0.82, 0.51], p >  0.05) or 1 hour (SMD: –0.18 [95% CI: –1.09, 0.74], p >  0.05) post-CWI, narrative synthesis suggested longer-term benefits, including a 29% reduction in sickness absence among participants who took cold showers. Improvements were also observed in sleep quality and quality of life, but not mood.

**Conclusions:**

This systematic review suggests that CWI delivers time-dependent effects on inflammation, stress, immunity, sleep quality, and quality of life, offering potential practical applications for health practitioners considering CWI for stress management and wellbeing support. However, the current evidence base is constrained by few RCTs, small sample sizes, and a lack of diversity in study populations. Future high-quality RCTs are needed to examine the long-term effects of CWI, its impact on diverse health outcomes, and optimal CWI protocols.

PROSPERO (ID: CRD42024500591)

## Introduction

The therapeutic practice of cold-water immersion (CWI), such as ice baths or cold showers, has gained considerable traction in recent years as a potential modality for promoting overall health and wellbeing [1]. CWI, which involves immersing the body partially or fully in cold water, in temperatures typically ranging from 10–15°C (50–59°F) [2,3], has been utilized across various cultures for centuries, but it is only in the modern era that it has been promoted as a remedial and performance-enhancing therapy in sport settings and, more recently, as a regular addition to self-care routines among the general population. CWI is believed to elicit a range of physiological responses, including the activation of the autonomic nervous system, modulation of the immune system, and the release of various biochemical mediators [2–5]. These physiological effects have prompted investigations into the potential applications of CWI in managing a diverse array of health conditions and promoting overall wellness.

CWI has been extensively researched in sporting contexts, with a notable emphasis on its ability to accelerate recovery after exercise. Meta-analysis has demonstrated that CWI after strenuous exercise can speed up the recovery of physical function, reduce muscle soreness, enhance perceived feelings of recovery, and reduce post-exercise inflammation [6]. However, the ability to accelerate acute recovery post-exercise may have long-term negative implications for desirable training outcomes. Most notably, there is a growing body of evidence suggesting that the regular use of CWI after training sessions can blunt increases in muscle size [7] and the development of muscular power and muscular strength [2,8]. While these findings underscore the nuanced applications of CWI in sports, much less is known about its potential effects and appropriate use among the general population, where its popularity is rapidly increasing. Despite this growing popularity, evidence supporting CWI’s broader health benefits remains limited and presents several important gaps. While studies have demonstrated that CWI can acutely increase heart rate and blood pressure while also causing significant increases in respiration rate and oxygen uptake [9], along with increased secretion of cortisol and norepinephrine [10], the long-term implications of these responses remain unclear. These physiological changes mirror exercise-induced responses, leading to speculation about potential cardiovascular health and cognitive benefits [11]. Similarly, while some evidence suggests that CWI can acutely improve mood [12], this finding is based on a small, non-randomized controlled trial where participants completed CWI in a group setting at the beach, raising questions about the role of social interaction and the natural environment in these effects. Proposals for using CWI to treat mood disorders such as depression [13], are further hampered by significant methodological limitations in existing studies. Research examining CWIs effects on physiological and mental health often combines it with physical activity (i.e., cold water swimming) or compares regular cold-water users (i.e., winter swimmers) to dissimilar control groups without accounting for lifestyle factors such as diet and exercise [14]. These confounding variables make it difficult to isolate the specific effects of CWI and draw reliable conclusions about its effectiveness.

These gaps and limitations are particularly noteworthy when considering the rapid rise that CWI has experienced amongst the general population in recent years. In common media, CWI has been purported to improve physical health and mental wellbeing, reduce inflammation, increase metabolic rate, and even enhance focus and cognition [15]. Recent reports have further highlighted this increase in popularity, with ice bath sales on the online store Amazon increasing from less than 1000 units in November 2022 to over 90,000 units 12 months later in November 2023 [16]. The disconnect between this growing popularity and the limited quality evidence supporting many claimed benefits underscores the need for more rigorous investigation.

This systematic review and meta-analysis aimed to summarize the available literature on the effects of CWI in the general population on various aspects of health and wellbeing, including psychological (mental wellbeing, depression, anxiety, stress, mood), cognitive (concentration, alertness, focus), and physiological (stress, fatigue, sleep, energy, skin health, immunity) outcomes, while critically evaluating the quality and limitations of existing evidence.

## Methods

### Design

This review followed the Preferred Reporting Items for Systematic Reviews and Meta-Analyses (PRISMA) statement for the reporting of systematic reviews and meta-analyses [17]. The protocol for this systematic review and meta-analysis was prospectively registered on PROSPERO (ID: CRD42024500591).

### Deviations from protocol

During screening, further eligibility criteria were added to standardize study conditions. Inclusion criteria of CWI immersion depth to at least chest level was added due to varying immersion methods, including single limb, waist, and chest level. Exclusion criteria of wearing protective garments such as wetsuits during CWI was added. Excluding studies that used protective garments ensured that the study measured the direct effects of cold water on the body without any insulation. The protocol was also revised to include inflammation as an outcome, which was initially excluded.

### Search strategy and selection criteria

In a cross-disciplinary approach, we identified ten databases in which to perform the search: CINAHL, Cochrane, MEDLINE, EmCare, ProQuest Health and Medical Collection, ProQuest Nursing and Allied Health, PsycINFO, Scopus, SPORTDiscus, and Web of Science. All databases were searched up until 17^th^ January 2024, using subject heading, keyword, and MeSH term searches for terms related to “cold-water immersion” (see S1 File for the full search strategy). A broad search strategy was used to capture as many studies as possible reporting outcomes related to CWI.

Database search results were exported to EndNote (version 20.2.1), where duplicates were removed and then uploaded to Covidence (Veritas Health Innovation, Melbourne, Australia). Two independent reviewers screened all titles and abstracts, and full-text articles for eligibility; any discrepancies were resolved by a third independent reviewer.

Inclusion criteria were as follows: i) healthy adults aged ≥ 18 years, ii) acute or long-term exposure to CWI, iii) CWI exposure was conducted via cold shower, ice bath, or cold plunge, iv) CWI water temperature was ≤ 15°C, v) a minimum CWI exposure time of 30 seconds, vi) immersion was at or above chest level (defined as the xiphoid process), vii) reported outcomes related to sleep, stress, fatigue, energy, skin health, immunity, inflammation, mental wellbeing, depression, anxiety, mood, concentration, alertness, or focus, viii) randomized controlled trial, and ix) published in a peer-reviewed journal. Studies were excluded if they included i) athletes, tier 3 and above [18], which includes highly trained and elite athletes competing at a national level or higher, ii) populations with chronic illness or musculoskeletal injury, iii) CWI exposure was through cryotherapy chamber or accidental exposure, iv) participants wore protective garments. CWI interventions that were conducted pre- or post-exercise were included if they reported appropriate outcomes.

### Quality assessment

Studies were critically appraised for methodological quality by two independent authors using the PEDro scale [19]. Any discrepancies were resolved by an independent author. The PEDro scale assesses eligibility criteria, random allocation, concealed allocation, baseline comparability, blinding of participants, therapists and assessors, adequate follow-up, intention-to-treat, between-group comparison, and point measures and variability. Each appraisal item was given a score of ‘yes,’ ‘no,’ or ‘not reported.’ A score ≥ 7 was considered ‘high quality,’ a score of 5 or 6 was considered ‘moderate quality,’ and ≤ 4 was considered ‘poor quality.’

### Data extraction

Data were extracted independently by two authors using a pre-determined data extraction template and performed in Covidence (Veritas Health Innovation, Melbourne, Australia). Data extracted included publication details (author information, publication date, country of origin, funding), study methodology (sample size, study type, timepoints, intervention conditions), participant information (age, sex, health status, physical activity level), CWI protocol (temperature, duration, number of immersions, depth of immersion, timing of immersion), comparator or control protocol, and outcome measures (scores, values). Discrepancies regarding data extraction were resolved by consensus with a third author.

### Data synthesis

Meta-analyses were performed to assess the effects of CWI on outcomes that were reported in at least two studies. For each meta-analysis, data were combined at the study level using a random effects model. A random effects model was used for all analyses to account for heterogeneity across studies, including variations in populations, interventions, and outcome measures, ensuring a more robust and conservative estimation of the pooled effect size [20]. Outcomes of interest were analyzed as continuous variables, and data were pooled using means and standard deviations (SDs) of pre-CWI exposure versus i) immediately post; ii) 1 hour post; iii) 12 hours post; iv) 24 hours post; v) 48 hours post; and vi) 30 days post-CWI exposure. Standardized mean differences (SMDs) were used as the effect measure for meta-analyses to allow comparison of data from different scales. All SMDs were calculated using RevMan software (version 5, Cochrane Collaboration). Each outcome was analyzed independently; the grouping of outcomes in forest plots reflects the software’s organizational structure and should not be interpreted as subgroup analyses.

If means and SDs were not reported in a study, authors were contacted, and data were requested. If the authors did not respond, then means and SD were calculated based on available data using recommended formulas (e.g., using sample size, median, and range) [21], or means and SDs were estimated using Plot Digitizer (https://plotdigitizer.com/) for data that was reported in graphical format only. If post-CWI exposure SDs were not reported or able to be estimated, then baseline SDs (rather than the SD of change scores) were used to avoid inflating effect sizes. When multiple intervention groups were compared to a single control group, we divided the “shared” control group sample size by the number of intervention groups to ensure appropriate weighting and transparency in our analyses. All meta-analyses were performed using RevMan software (version 5, Cochrane Collaboration). For outcomes not suitable for meta-analysis, narrative synthesis was conducted. Outcomes were synthesized narratively if they were: i) reported in single studies only; ii) measured at substantially different timepoints across studies; or iii) assessed using outcome measures that prevented statistical pooling.

Publication bias was evaluated using funnel plots of SMDs and standard errors and evaluating for asymmetries or missing sections within the plot [20]. The Cochran’s Q test was used to assess statistical heterogeneity and the I^2^ statistic to quantify the proportion of the overall outcome attributed to variability [20]. The following cut-off values for the I^2^ statistic were used: 0 to 29% =  no heterogeneity; 30 to 49% =  moderate heterogeneity; 50 to 74% =  substantial heterogeneity; and 75 to 100% =  considerable heterogeneity [20]. Standardized classifications for the magnitude of effect were used (0.20 =  small effect; 0.20 to 0.50 =  medium effect; and greater than 0.50 =  large effect) [22]. A p-value of <  0.05 was considered statistically significant.

The certainty of evidence was graded using the Oxford Centre for Evidence-Based Medicine 2011 Levels of Evidence [23], as follows: grade A: consistent level 1 studies (i.e., individual RCTs); B: consistent level 2 (i.e., individual cohort studies) or level 3 studies (i.e., individual case-control studies) or extrapolations from level 1 studies; C: level 4 studies (i.e., case series) or extrapolations from level 2 or 3 studies; or D: level 5 (i.e., expert opinion without explicit critical appraisal) evidence or inconsistent or inconclusive studies of any level [24].

## Results

### Search results

**Fig 1:**
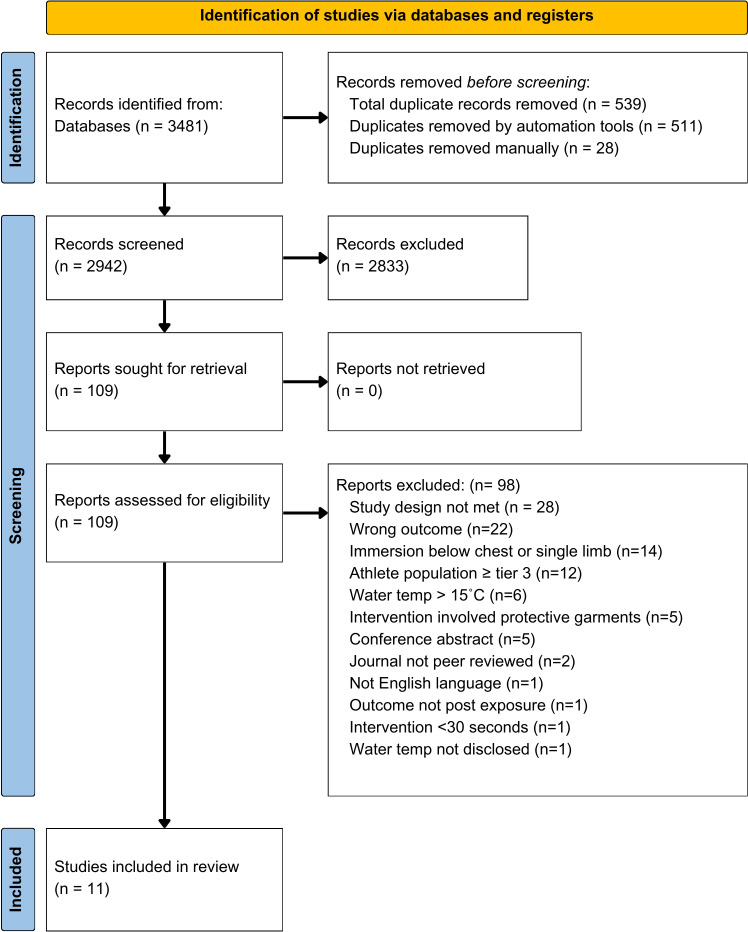
PRISMA flowchart for screening of articles.

The database search identified 3481 studies, of which 109 were eligible for full-text review. Of these articles, 98 were excluded: 70 did not meet the inclusion criteria, and 28 did not meet study design criteria. A total of 11 studies were included (Fig 1). Further information was sought from 7 studies [5,25–30] due to graphical representation of data. We received one response from the author [30] on the 26^th^ of March 2024. Plot Digitizer was used to extract graphical data from 5 studies [5,26–29]. Data was not obtained for one study [25].

### Study characteristics

Included studies were published between 2014 and 2023 and were conducted in Australia [25–27,29–32], Lithuania [5,28,33,34], the Netherlands [33], Switzerland [32], Japan [29], the United States [26], and Finland [25]. These studies comprised a total of 3177 participants, of whom 55.4% were female. The provision of female participants was provided by one study [33], comprised of 3018 participants. All other studies were comprised of 100% male participants. Study design was varied; six studies [5,25–27,29,30] were conducted in a randomized crossover design, and 5 studies [28,31–34] were conducted in a randomized parallel design. Intervention consistencies were seen across the studies. Ten out of eleven studies reported CWI interventions performed in a bath, with only one study [33] reporting to have used cold showers. Ten out of eleven studies reported seated and/or no CWI as the comparator or control method; only one study [30] reported active recovery as a comparator. Other intervention characteristics were varied. Water temperature ranged from 7 [32] to 15°C [26,29], and immersion time varied between 30 s [33] and 2 hours [27]. A summary of study characteristics is shown in Table 1.

**Table 1:** Characteristics of the included studies.

Author, year	Country	Study design	Participants (N)	Age	Female (%)	Intervention type	CWI^a^ temperature (°C)	CWI duration	Depth of immersion	Comparator/Control
Ahokas, 2020	Finland	Crossover	9	26 ± 3.7	0	Bath	10	10 min	Xiphoid process	Seated in an empty bath for 10 min^b^
Buijze, 2016	Netherlands	Parallel	798 (30 s) 727 (60 s) 775 (90 s) 718 (CON^c^)	39.7 ± 11.3 (30 s) 38.9 ± 10.6 (60 s) 39.6 ± 10.6 (90 s) 39.2 ± 10.6 (CON)	59 (30 s) 58 (60 s) 60 (90 s) 56 (CON)	Cold shower	10–12	30 consecutive days 30, 60, 90 s	Whole body	Shower as regular (not cold)
Earp, 2019	United States	Crossover	11	21.1 ± 2.1	0	Bath	15	15 min	Xiphoid process	Seated for 1 hour in similar body position to during CWI
Eimonte, 2021A	Lithuania	Parallel	16 (CWI) 10 (CON)	24 ± 1 (CWI) 24 ± 2 (CON)	0	Bath	14	20 min × 6 (10 min rest between EXP^d^)	Manubrium	Remained at ambient temperature of 24 °C, 60% relative humidity without CWI for 170 min
Eimonte, 2021B	Lithuania	Crossover	12	23 ± 4	0	Bath	14	10 min	Manubrium	Remained at ambient temperature of 24 °C, 60% relative humidity without CWI for 10 min
Eimonte, 2022	Lithuania	Crossover	17	24 ± 1	0	Bath	14	Long: Intermittent 170 min (120 min maximum total immersion time) Short: 10 min	Manubrium	Seated in a towel covered bath, at ambient temp 24 °C, 60% relative humidity, same duration as CWI: long and short
Hironaga, 2019	Japan	Crossover	7	22.9 ± 1.6	0	Bath	15	1 min in water, 1 min out of water x7	Shoulder	Sitting at rest for 14 min
Roberts, 2014	Australia	Crossover	10	21.3 ± 1.6	0	Bath	10 ± 0.3	10 min	Clavicle	Active recovery (Exercising on a cycle ergometer for 10 mins at a low, self-selected intensity)
Rose, 2023	Australia	Parallel	9 (CWI) 9 (CON)	23.4 ± 4.6 (total sample)	0	Bath	9	3 min	Xiphoid process	Seated quietly on a chair at room temperature of 24 °C for 3 min
Skein, 2018	Australia	Parallel	10 (CWI) 10 (No CWI)	22 ± 2 (CWI) 24 ± 5 (No CWI)	0	Bath	14.1 ± 0.4	15 min	Sternal notch	No CWI
Versteeg, 2023	Switzerland	Parallel	6 (CWI) 6 (CON)	25.2 ± 4.0 (total sample)	0	Bath	7 ± 0.5	12 min	Sternum (arms out)	No cold exposure

^a^CWI, cold-water immersion.

^b^min, minutes.

^c^CON, control.

^d^EXP, exposure.

### Risk of bias

Assessment of the methodological quality of the studies using the PEDro scale produced a mean of 6.4. Seven studies [25–27,29–32] were considered as ‘moderate quality’ and another four studies [5,28,33,34] as ‘high quality’ (see S2 File for full results of the PEDro assessment).

### Meta-analysis results

There was sufficient data to perform meta-analyses for the following outcomes: i) inflammation (immediately and 1 hour post-CWI); ii) stress (immediately, 1 hour, 12 hours, 24 hours, and 48 hours post-CWI); and iii) immunity (immediately and 1 hour post-CWI).

### Inflammation

**Fig 2:**
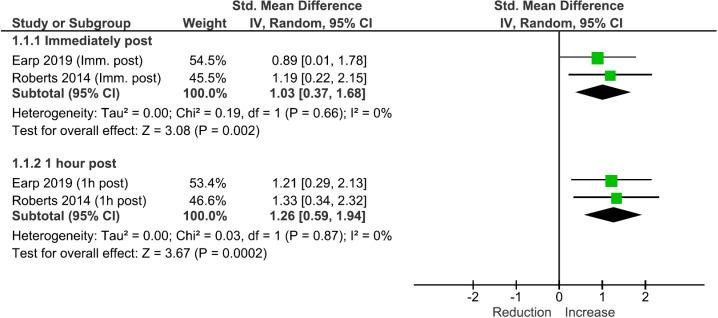
Meta-analysis results of the effects of cold-water immersion on inflammation immediately post and 1 hour post exposure. *IV* instrumental variables, *CI* confidence interval, *Imm.* immediately.

Results of meta-analysis showed a significant increase in inflammation immediately (SMD: 1.03 [95% CI: 0.37, 1.68], p <  0.01) and 1 hour post (SMD: 1.26 [95% CI: 0.59, 1.94], p <  0.01) CWI exposure (Fig 2). The overall certainty of evidence was Grade B.

### Stress

**Fig 3:**
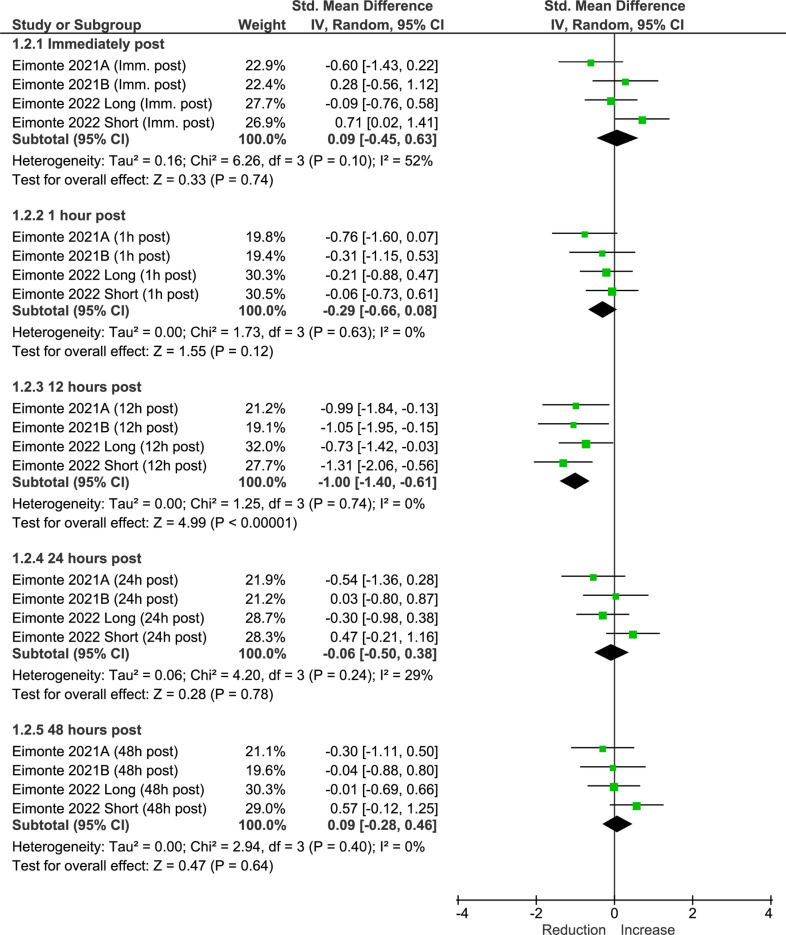
Meta-analysis results of the effects of cold-water immersion on stress immediately post, 1 hour post, 12 hours post, 24 hours post, and 48 hours post-exposure. *IV* instrumental variables, *CI* confidence interval, *Imm.* immediately.

Meta-analysis results showed no significant changes in stress immediately post (SMD: −0.09 [95% CI: −0.45, 0.63], p >  0.05), 1 hour post (SMD: −0.29 [95% CI: -0.66, 0.08], p >  0.05), 24 hours post (SMD: −0.06 [95% CI: -0.50, 0.38], p >  0.05) and 48 hours post (SMD: 0.09 [95% CI: −0.28, 0.46], p >  0.05) CWI exposure (Fig 3). A significant reduction in stress was observed 12 hours post CWI exposure (SMD: −1.00 [95% CI: −1.40, −0.61], p <  0.01). The overall certainty of evidence was Grade B.

### Immunity

**Fig 4:**
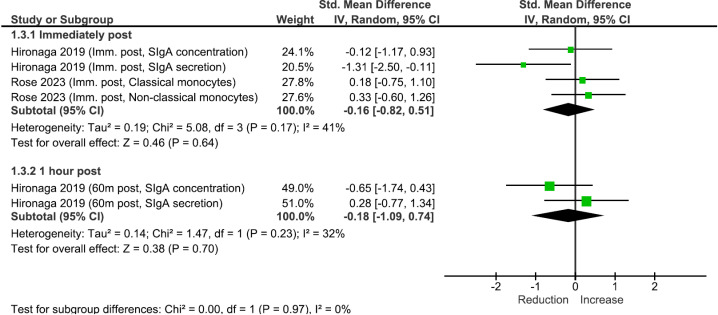
Meta-analysis results of the effects of cold-water immersion on immunity immediately post- and 1 hour post-exposure. *IV* instrumental variables, *CI* confidence interval, *Imm.* Immediately.

No significant changes in immunity were observed immediately post (SMD: −0.16 [95% CI: −0.82, 0.51], p >  0.05) or 1 hour post CWI exposure (SMD: -0.18 [95% CI: −1.09, 0.74], p >  0.05) (Fig 4). The overall certainty of evidence was Grade D (inconsistent or inconclusive studies of any level).

### Narrative syntheses

Narrative syntheses were conducted for outcomes reported in single studies or where the timing of assessments was considered too different to allow results to be meaningfully combined in a meta-analysis.

### Inflammation and immunity

Versteeg et al. (2023) evaluated the effects of a 3-week repeated CWI in a sample of 12 males randomly assigned to either CWI (12 minutes at 7°C, 4 times/week) or a control condition. While total leukocyte counts significantly decreased in both the CWI group (median difference: −1.10 ×  10^3^/μL) and the control group (median difference: −0.8 ×  10^3^/μL), the minimal reduction observed in both groups suggests that repeated CWI did not result in clinically relevant changes in inflammation. Ahokas (2020) compared the efficacy of CWI recovery (10°C for 10 minutes) to an active recovery control in random order among 9 physically active males, following an intensive loading protocol. A significant increase in leukocyte and lymphocyte concentrations (p <  0.01) occurred 5 minutes post protocol. At 1 hour post-recovery, leukocytes returned to baseline, and a significant drop below baseline was seen in lymphocytes (p <  0.001). Lymphocytes returned to baseline post 24 hours. Despite these results, no differences were seen for leukocyte and lymphocyte response between CWI and control groups. Both studies are limited by small sample sizes, homogeneous participant groups, and a lack of control for confounding variables such as baseline fitness. These factors restrict the generalizability of findings and may contribute to variability in reported outcomes. The lack of long-term follow-up further limits the ability to assess sustained effects of CWI on inflammation and immunity.

### Sickness and illness

In a pragmatic randomized controlled trial (n = 3018) by Buijze et al. (2016), participants aged 18–65 years were randomly assigned to CWI for either 30, 60, or 90 seconds, or to a control group, for 30 days. Findings showed a 29% reduction in sickness absence for those in any of the CWI groups compared to the control group (incident rate ratio: 0.71, P =  0.003). However, for the number of illness days reported, there was no significant difference between groups. The large sample size and pragmatic design improve the external validity of this study. However, the reliance on self-reported sickness absence introduces potential reporting and social desirability bias. The reduction in sickness absence without changes in illness days raises questions about the mechanisms driving these findings.

### Sleep

Sleep quality was assessed (1–5 Likert scale) by Skein (2018) in males without prior sleep conditions or altered sleep patterns. Participants were randomly assigned to CWI or passive recovery for 15 minutes following heat training over 5 consecutive days. Post intervention, sleep quality scores were better for the CWI groups (P =  0.04), with mean and SD scores of 3.6 ±  0.5 and 3.1 ±  0.5 for the CWI and passive recovery groups, respectively. While the findings suggest potential benefits of CWI on sleep quality, the exclusive inclusion of male participants limits broader applicability, and the effects may have been confounded by the post-heat training recovery context.

### Mood

An assessment of moods (modified Profile of Moods States) was undertaken by Skein (2018) in 20 male participants aged 24 ±  4 years who were randomly assigned to CWI and passive recovery groups. Post intervention, there was no significant difference seen for moods (active, energetic, restless, fatigued, exhausted, and alert) between the CWI and passive recovery groups.

### Quality of life

In a study with 3018 participants, those who took 30, 60, or 90-second cold showers for 30 days showed slightly higher median quality of life scores, assessed using the SF-36 survey, compared to the control group [33]. The median SF-36 mental component scores after 30 days were slightly higher for all cold shower intervention groups (84.7, interquartile range 76.4–90.2; 85.1, interquartile range 76.7–90.6; 85.7, interquartile range 78–90.8) compared to the control group (83.9, interquartile range 72.9–89.4; p <  0.01). However, these differences were no longer significant after 90 days. While the large sample size strengthens statistical power, the study faced significant methodological challenges, particularly regarding intervention adherence over time. The loss of significance at 90 days raises questions about participant compliance. Additionally, the self-reported nature of the SF-36 survey, combined with the inability to blind participants to the intervention, may have introduced reporting bias, including the potential for social desirability bias.

## Discussion

CWI has gained widespread popularity among the general public, despite most evidence focusing solely on its effects among athletic populations. This knowledge gap is particularly concerning given the increase in consumer adoption of CWI, with largely unsupported claims in mainstream media about its benefits for physical health and mental wellbeing. This systematic review and meta-analysis aimed to evaluate the health and wellbeing effects of CWI among healthy adults, focusing on various psychological, cognitive, and physiological outcomes. We analyzed 11 studies with a total of 3,177 participants. Our key findings showed a significant increase in inflammation immediately and 1 hour after CWI, suggesting an acute inflammatory response. Interestingly, we observed a significant reduction in stress 12 hours post-CWI, although no significant effects on stress were detected immediately, 1 hour, 24 hours, or 48 hours post-exposure. While the meta-analysis revealed no significant effects on immune function immediately or 1 hour post-CWI, the narrative synthesis suggested longer-term benefits, with a 29% reduction in sickness absence among participants who took cold showers. Improvements were also observed in sleep quality and quality of life, while no significant differences were found in mood.

Our findings of short-term increased inflammation contrast with a previous review that reported reductions in inflammation following CWI, such as decreased tumor necrosis factor-α 24 hours post-immersion [35]. However, our findings are consistent with another review, which reported higher interleukin-6 levels in longer 30-minute CWI protocols [36], indicating increased inflammation. Immediate increases in inflammation markers may reflect metabolic responses such as glycogenolysis rather than systemic inflammation, while measurements taken 24 hours post-CWI likely reflect reduced muscle temperature and metabolic activity, leading to decreases in systemic inflammation [30]. Differences in biomarkers used to test for inflammation may also contribute to this variability [30].

To our knowledge, no comparative systematic reviews on CWIs effect on immunity exist. A limited amount of previous exploratory evidence shows mixed results on the effects of CWI on immunity, likely influenced by variations in cooling protocols, individual physiological responses, and specific markers of innate and adaptive immunity assessed. One study observed that a short CWI protocol (12 minutes at 10°C) post-endurance exercise increased neutrophil counts immediately post, but it did not impact lymphocytes, monocytes, or systemic inflammatory markers 24 or 48 hours post-exposure [37]. And another study found that CWI could stimulate innate immunity by increasing neutrophils and IL-6 while suppressing adaptive immunity through reductions in lymphocytes and monocytes [11]. These findings highlight the complexity of immune responses to CWI, driven by differences in protocols, physiological adaptations, and environmental factors.

The temporal pattern of stress responses to CWI reveals a complex relationship between immediate activation and delayed adaptation. The initial “cold shock” response is characterized by rapid sympathetic nervous system activation, with significant increases in norepinephrine levels and systolic blood pressure within 2–15 minutes of immersion [38], though notably without substantial epinephrine elevation [38,39]. This immediate stress response contrasts with the delayed effects observed 12 hours post-CWI, when stress markers show significant reduction [5]. The hypothalamic-pituitary-adrenal axis involvement appears more nuanced than previously thought; while some studies show increases in cortisol during cold exposure [39], controlled CWI can produce modest cortisol responses [38]. The gradual transition from sympathetic to parasympathetic dominance is mediated through baroreceptor feedback mechanisms [38], leading to delayed effects on inflammatory markers such as IL-6 and TNF-α [5]. This stress-regulatory pattern is notably influenced by immersion protocols, with shorter exposures (≤10 minutes) primarily triggering acute sympathetic responses [5,39], while longer or repeated exposures may facilitate adaptive mechanisms [5]. Briganti et al.‘s (2023) systematic review particularly emphasizes how these varied outcomes highlight the importance of standardized protocols for achieving consistent therapeutic benefits [39].

Despite observing improvements in stress, our review found no significant differences in mood immediately after CWI. However, it is important to note that only one study in our review examined mood. Its findings, reached under RCT conditions, contrast with findings from a non-RCT (which was not eligible for our review), which suggested that CWI improves mood by way of reduced negative mood sub-scales and increased positive ones in young, healthy individuals [12]. It is possible that differences in findings between these two studies were due to differences in the instruments used to measure mood. Tools like the Positive and Negative Affect Schedule (PANAS) and the Global Mood Scale (GMS) differ in their conceptualization of ‘negative affect,’ with PANAS focusing on anxiety and GMS on exhaustion [40]. This highlights how varied frameworks can influence outcomes.

Behavioral and quality of life outcomes showed promising results in the systematic review. Improvements were noted in these areas, although the lack of comparative studies on CWIs effect on quality of life highlights a significant gap in the literature. The Wim Hof Method, which includes cold exposure, specific breathing exercises, and meditation, has similarly demonstrated quality of life benefits [41]. However, the combined effects of its various components make it difficult to isolate the specific contribution of CWI. Studies with athlete populations have examined the impact of CWI on sleep. One such study [42] found that CWI after high-intensity exercise did not acutely affect overall night sleep quantity and quality, suggesting that CWI does not impact sleep when used post-exercise. Another study [43] indicated that whole and partial CWI post-exercise decreased sleep arousals and limb movements, particularly benefiting athletes during intense training and competition periods. Overall, CWI shows promise for improving sleep and quality of life, although its specific contributions are hard to isolate.

A key strength of our review is its novelty; to our knowledge, it is the first systematic review of general and wide-ranging health impacts of CWI in general populations. It considerably extends previous systematic reviews, which have considered the impacts of CWI in the context of exercise performance [2,6,44–46], muscle soreness [46–48], and athlete populations [49,50]. We adhered to the highest quality systematic review methodology, including a comprehensive search strategy across multiple databases, ensuring a broad capture of relevant studies. The use of rigorous inclusion criteria and the restriction to RCTs enhance the quality and validity of our findings. Furthermore, we used a meta-analysis synthesis approach for all outcomes with sufficient data to enable it. Several limitations also exist. The studies varied widely in their CWI protocols, likely contributing to heterogeneity in the results. Six of the eleven studies examined the impact of single immersions, preventing conclusions on the long-term effects of CWI. Only one study included female participants, limiting the applicability of results to females. Five studies implemented CWI following exercise, potentially confounding the effects with post-exercise recovery processes. Small sample sizes in many of the included studies impact the generalizability of the results. The prevalence of moderate to high-quality studies strengthens confidence in our findings, particularly for outcomes where multiple studies were meta-analyzed. However, some outcomes, including inflammation and immunity, sleep, and mood, represented in the narrative synthesis were derived from single moderate-quality studies, suggesting their findings should be interpreted more cautiously.

This review highlights that there is currently limited high-quality evidence on the impacts of CWI for the general population. Early evidence suggests that CWI has significant impacts on inflammation, stress, immunity, quality of life, and sleep. However, the impacts of CWI also appeared highly time-dependent, with different impacts on stress, inflammation, and immunity observed immediately following CWI, across the day after CWI, and at longer-term follow-up. Despite these findings, the application of CWI in real-world or clinical settings for the general population remains limited by the variability in protocols and the heterogeneity of study populations. The highly time-dependent nature of CWIs effects suggests that tailored approaches are necessary to explore its potential benefits. For example, the delayed reduction in stress observed 12 hours post-immersion may make CWI a valuable aid to stress management strategies in healthy individuals. Similarly, improvements in sleep quality and sickness absence suggest that CWI could be investigated as a complementary approach for enhancing general wellbeing. However, the lack of long-term data, the reliance on single-session studies, and the small sample sizes in many trials highlight the need for further research before CWI can be widely recommended.

Given the high public interest and potential risks of CWI, more research is needed to understand its benefits and risks. High-quality RCTs are needed that:

1. Examine long-term effects: Most studies to date have focused on the acute effects of a single CWI. RCTs with repeated CWI and longer-term follow-up are needed to evaluate the sustained effects of CWI on health outcomes.
2. Large and diverse samples: RCTs with larger samples are needed to enhance statistical power and the reliability of findings. Including more diverse populations in terms of age, gender, and health status will improve the generalizability of findings. Studies examining the effects of CWI in individuals with specific health conditions, such as autoimmune disorders and mental health disorders, are also warranted.
3. Diverse outcomes: Measure the potential behavioral, physical, mental health, and cognitive impacts of CWI using validated measures. This may include outcomes such as sleep, mood, anxiety, depression, cognitive function, and weight-related outcomes.
4. Dose-response relationships: Investigate the dose-response aspects of CWI, including the optimal temperature, duration, and frequency of exposure. Additionally, examine CWI combined with other interventions (such as exercise or the Wim Hof Method) to understand the additive or synergistic effects.

Though beyond the scope of our review, which has focused on effectiveness, future studies should also closely examine safety and participant experience, assessing the subjective experiences of participants undergoing CWI to identify factors that influence adherence and satisfaction.

In conclusion, this systematic review and meta-analysis provides the first comprehensive evaluation of the health and wellbeing effects of CWI among healthy adults. Our analysis of 11 studies involving 3,177 participants revealed several potential benefits, including a reduction in stress 12 hours post-CWI, with improvements also noted in sleep quality and quality of life. However, purported benefits remain unsubstantiated, with inconclusive evidence regarding CWIs impact on immunity and mood, and concerning findings of short-term increases in inflammation. The highly time-dependent nature of CWIs effects further complicates the risk-benefit assessment. These mixed findings, coupled with growing public interest in CWI, underscore the need for caution and additional research. Future high-quality RCTs with larger, more diverse samples are essential to fully understand both the therapeutic potential and safety concerns. This includes examining long-term effects, diverse health outcomes, and dose-response relationships, as well as monitoring adverse effects and subjective experiences. Overall, while CWI shows promise for specific outcomes, more robust evidence is required to establish its safety profile and validate its purported health benefits.

## Supporting information

S1 File Search strategy. (PDF)

S2 File Pedro assessment. (PDF)

S3 File CWI protocol. (PDF)

S4 File Prisma checklist. (PDF)

S5 File Analysis data. (XLSX)

S6 File Data extraction. (XLSX)

S7 File Included studies. (XLSX)

S8 File Excluded studies. (XLSX)

S9 File PICO. (PDF)

S10 Fig Funnel plot of comparison inflammation (acute). (TIF)

S11 Fig Funnel plot of comparison for stress (acute). (TIF)

S12 Fig Funnel plot of comparison for immunity. (TIF)

S13 Fig Funnel plot of comparison for quality of life (30 days). (TIF)
