# Supplementary material for: Effects of cold-water immersion on health and wellbeing: A systematic review and meta-analysis
Source: PLoS One. 2025 Jan 29;20(1):e0317615. doi: 10.1371/journal.pone.0317615 (PMC11778651; doi:10.1371/journal.pone.0317615)
Supplement: S1 File — (PDF) [file pone.0317615.s001.pdf]

Search strategy

This systematic review search strategy will be conducted in line with the Preferred Reporting Items for Systematic reviews and Meta-Analyses (PRISMA) 2020 statement. Selected studies will be limited to randomised controlled trials which investigated the effects of CWI associated with health, well-being and cognitive outcomes in humans. Observational studies, quasi-experimental studies, reviews, expert opinions, commentaries, letters to editor, studies on animals, and conference abstracts will be excluded. Searches will use search terms related to CWI, study type and exclusion criteria (Table 2). No year restriction will be placed on the search. To identify additional articles for possible data, reference lists of all selected studies will be screened for potentially relevant articles.

Table 2. Search terms

Search Category	Search Terms
Cold-water immersion	(cold immersion) OR (cold water immersion) OR (cold-water immersion) OR (cold water exposure) OR (cold-water exposure) OR (ice water immersion) OR (ice-water immersion) OR (ice bath) OR (ice-bath) OR (cold shower) OR (cold showering) OR (cold plunge) OR (cold-plunge)
Study type	AND (RCT) OR (random* control* trial*) OR (random* control* study) OR (control* trial*)
Exclusion criteria	NOT (athlete*)
