# Supplementary material for: Effects of cold-water immersion on health and wellbeing: A systematic review and meta-analysis
Source: PLoS One. 2025 Jan 29;20(1):e0317615. doi: 10.1371/journal.pone.0317615 (PMC11778651; doi:10.1371/journal.pone.0317615)
Supplement: S2 File — (PDF) [file pone.0317615.s002.pdf]

Author, year	Eligibility criteria	Random allocation	Concealed allocation	Baseline comparability	Blinding of participants	Blinding of therapists	Blinding of assessors	Adequate follow-up	Intention to treat	Between-group comparison	Point measures and measures of variability	Score
**Ahokas 2020**	No	Yes	Not reported	Yes	Not reported	Not reported	Not reported	Yes	Yes	Yes	Yes	6
**Buijze 2016**	Yes	Yes	Yes	Yes	No	Not reported	Not reported	No	Yes	Yes	Yes	7
**Earp 2019**	No	Yes	Not reported	Yes	Not reported	Not reported	Not reported	Yes	Yes	Yes	Yes	6
**Eimonte 2021**	Yes	Yes	Not reported	Yes	Not reported	Not reported	Not reported	Yes	Yes	Yes	Yes	7
**Eimonte 2021**	Yes	Yes	Not reported	Yes	Not reported	Not reported	Not reported	Yes	Yes	Yes	Yes	7
**Eimonte 2022**	Yes	Yes	Not reported	Yes	Not reported	Not reported	Not reported	Yes	Yes	Yes	No	6
**Hironaga 2019**	Not reported	Yes	Not reported	Yes	Not reported	Not reported	Not reported	Yes	Yes	Yes	Yes	6
**Roberts 2014**	No	Yes	No	Yes	No	No	Not reported	Yes	Yes	Yes	Yes	6
**Rose 2023**	Yes	Yes	Not reported	Yes	No	No	Not reported	Yes	Yes	Yes	Yes	7
**Skein 2018**	Yes	Yes	Not reported	Yes	Not reported	Not reported	Not reported	Not reported	Yes	Yes	Yes	6
**Versteeg 2023**	Yes	Yes	Not reported	Yes	No	Not reported	Not reported	Not reported	Yes	Yes	Yes	6
