# Supplementary material for: Effects of cold-water immersion on health and wellbeing: A systematic review and meta-analysis
Source: PLoS One. 2025 Jan 29;20(1):e0317615. doi: 10.1371/journal.pone.0317615 (PMC11778651; doi:10.1371/journal.pone.0317615)
Supplement: S3 File — (PDF) [file pone.0317615.s003.pdf]

National Institute for Health Research

University of York
Centre for Reviews and Dissemination

Systematic review

A list of fields that can be edited in an update can be found here

1. * Review title.

Give the title of the review in English

Effects of cold-water immersion on health and wellbeing: A systematic review and meta-analysis

2. Original language title.

For reviews in languages other than English, give the title in the original language. This will be displayed with the English language title.

3. * Anticipated or actual start date.

Give the date the systematic review started or is expected to start.

23/01/2024

4. * Anticipated completion date.

Give the date by which the review is expected to be completed.

31/05/2024

5. * Stage of review at time of this submission. [1 change]

This field uses answers to initial screening questions. It cannot be edited until after registration.

Tick the boxes to show which review tasks have been started and which have been completed.

Update this field each time any amendments are made to a published record.

The review has not yet started: No

Review stage	Started	Completed
Preliminary searches	Yes	Yes
Piloting of the study selection process	Yes	Yes
Formal screening of search results against eligibility criteria	Yes	Yes
Data extraction	Yes	Yes
Risk of bias (quality) assessment	Yes	Yes
Data analysis	Yes	Yes

Provide any other relevant information about the stage of the review here.

6. * Named contact.

The named contact is the guarantor for the accuracy of the information in the register record. This may be any member of the review team.

Tara Cain

Email salutation (e.g. "Dr Smith" or "Joanne") for correspondence:

Mrs Cain

7. * Named contact email.

Give the electronic email address of the named contact.

caits001@mymail.unisa.edu.au

8. Named contact address

Give the full institutional/organisational postal address for the named contact.

Corner of North Terrace and, Frome Rd, Adelaide SA 5001

9. Named contact phone number.

Give the telephone number for the named contact, including international dialling code.

1300 301 703

10. * Organisational affiliation of the review.

Full title of the organisational affiliations for this review and website address if available. This field may be

completed as 'None' if the review is not affiliated to any organisation.

University of South Australia

Organisation web address:

https://www.unisa.edu.au/

11. * Review team members and their organisational affiliations.

[1 change]

Give the personal details and the organisational affiliations of each member of the review team. Affiliation refers to groups or organisations to which review team members belong. **NOTE: email and country now MUST be entered for each person, unless you are amending a published record. PLEASE USE AN INSTITUTIONAL EMAIL ADDRESS IF POSSIBLE.**

Mrs Tara Cain. University of South Australia
Professor Carol Maher. University of South Australia
Dr Ben Singh. University of South Australia
Dr Jacinta Brinsley. University of South Australia
Dr Hunter Bennett. University of South Australia
Dr Max Nelson. Universtity of South Australia

12. * Funding sources/sponsors.

Details of the individuals, organizations, groups, companies or other legal entities who have funded or sponsored the review.

This study is funded by an undergraduate scholarship provided by the Alliance for Research in Exercise, Nutrition and Activity (ARENA) Research Centre, at the University of South Australia.

Grant number(s)

State the funder, grant or award number and the date of award

13. * Conflicts of interest.

List actual or perceived conflicts of interest (financial or academic).

None

14. Collaborators.

Give the name and affiliation of any individuals or organisations who are working on the review but who are not listed as review team members. **NOTE: email and country must be completed for each person, unless you are amending a published record.**

15. * Review question.

State the review question(s) clearly and precisely. It may be appropriate to break very broad questions down into a series of related more specific questions. Questions may be framed or refined using PI(E)COS or

similar where relevant.

What effect does cold water immersion have on physiological, psychological and cognitive responses related to health and well-being in healthy adults?

16. * Searches.

State the sources that will be searched (e.g. Medline). Give the search dates, and any restrictions (e.g. language or publication date). Do NOT enter the full search strategy (it may be provided as a link or attachment below.)

A cross disciplinary approach will be applied to digital literature searches across Allied and Complementary Medicine (AMED), CINAHL, Cochrane, MEDLINE, Emcare, ProQuest Health and Medical Collection, ProQuest Nursing and Allied Health, APA PsycINFO, Scopus, Sport Discus, EBSCOhost and Web of Science databases. Searches will be conducted during January 2024. No date restriction will be placed on the search.

17. URL to search strategy.

Upload a file with your search strategy, or an example of a search strategy for a specific database, (including the keywords) in pdf or word format. In doing so you are consenting to the file being made publicly accessible. Or provide a URL or link to the strategy. Do NOT provide links to your search **results**.

https://www.crd.york.ac.uk/PROSPEROFILES/500591_STRATEGY_20240108.pdf

Alternatively, upload your search strategy to CRD in pdf format. Please note that by doing so you are consenting to the file being made publicly accessible.

Do not make this file publicly available until the review is complete

18. * Condition or domain being studied.

Give a short description of the disease, condition or healthcare domain being studied in your systematic review.

Health and well-being outcomes, encompassing psychological aspects such as mental well-being, depression, anxiety, stress and mood. Cognitive function including concentration, alertness, and focus, as well as physiological responses such as stress, fatigue, sleep, energy, skin health and immunity resulting from cold water immersion (CWI).

19. * Participants/population.

Specify the participants or populations being studied in the review. The preferred format includes details of both inclusion and exclusion criteria.

Healthy human participants aged ?18 years, with any level of experience of CWI will be included.

Populations consisting of professional athletes, have a chronic illness or musculoskeletal injury will be

excluded.

20. * Intervention(s), exposure(s).

Give full and clear descriptions or definitions of the interventions or the exposures to be reviewed. The preferred format includes details of both inclusion and exclusion criteria.

Intervention should be acute or long-term exposure to CWI in water ?15deg for a minimum of 30 seconds. CWI can be any time of day including pre or post exercise. Types of CWI to be included will be cold showers, ice baths or cold plunge. Cryotherapy (i.e. cold air therapy) and accidental exposure will be excluded.

21. * Comparator(s)/control.

Where relevant, give details of the alternatives against which the intervention/exposure will be compared (e.g. another intervention or a non-exposed control group). The preferred format includes details of both inclusion and exclusion criteria.

All comparators will be eligible.

22. * Types of study to be included.

Give details of the study designs (e.g. RCT) that are eligible for inclusion in the review. The preferred format includes both inclusion and exclusion criteria. If there are no restrictions on the types of study, this should be stated.

Randomised control trials, published in peer-reviewed sources (full length conference reports will be included) will be included. Observational studies, quasi-experimental studies, reviews, expert opinions, commentaries, letters to editor, studies on animals, and conference abstracts will be excluded.

23. Context.

Give summary details of the setting or other relevant characteristics, which help define the inclusion or exclusion criteria.

Studies reporting outcomes related to muscle soreness, muscle function, sports performance, athlete recovery, HR variability or inflammation will be excluded.

24. * Main outcome(s).

Give the pre-specified main (most important) outcomes of the review, including details of how the outcome is defined and measured and when these measurement are made, if these are part of the review inclusion criteria.

Any physiological, psychological or cognitive response related to sleep, stress, fatigue, energy, skin health, immunity, mental well-being, depression, anxiety, stress, mood, concentration, alertness, or focus.

Measures of effect

Please specify the effect measure(s) for you main outcome(s) e.g. relative risks, odds ratios, risk difference, and/or 'number needed to treat.

Any effect measures of relevance will be included.

25. * Additional outcome(s).

List the pre-specified additional outcomes of the review, with a similar level of detail to that required for main outcomes. Where there are no additional outcomes please state 'None' or 'Not applicable' as appropriate to the review

None

Measures of effect

Please specify the effect measure(s) for you additional outcome(s) e.g. relative risks, odds ratios, risk difference, and/or 'number needed to treat.

26. * Data extraction (selection and coding).

Describe how studies will be selected for inclusion. State what data will be extracted or obtained. State how this will be done and recorded.

Initial search results will be imported into Endnote by an individual researcher and duplicates will be removed. Covidence's AI RCT filter will be used to remove non-RCTs. Two independent researchers will screen remaining studies utilising Covidence. Initial pass will involve screening titles and abstracts; any irrelevant material will be excluded. Second pass will involve examining full text of remaining studies for compliance with eligibility criteria.

27. * Risk of bias (quality) assessment.

State which characteristics of the studies will be assessed and/or any formal risk of bias/quality assessment tools that will be used.

All studies included will be critically appraised for risk of bias. This process will be performed by two independent researchers using the PEDro scale. Each study will be assessed for specified eligibility criteria, random allocation, concealed allocation, baseline comparability, blinding of participants, therapists and assessors, measures of outcomes, intention-to-treat, between-group comparison, point measures and variability.

28. * Strategy for data synthesis.

Describe the methods you plan to use to synthesise data. This **must not be generic text** but should be **specific to your review** and describe how the proposed approach will be applied to your data. If meta-analysis is planned, describe the models to be used, methods to explore statistical heterogeneity, and software package to be used.

Meta-analyses will be used to pool the results for all outcomes of interest. We will report effect sizes and 95% confidence intervals for outcomes in included studies. Forest plots will display standardized mean differences and corresponding 95% CI, with Hedges' g used for assessing statistical significance. Effect magnitudes will be categorised as follows: 0.20, negligible; 0.20 to 0.49, small; 0.50 to 0.79, moderate; ?0.80, large. Cochran's Q test and I^2 will evaluate statistical heterogeneity. A random-effects model will be

applied for meta-analysis of heterogeneous studies. Insufficient data for meta-analysis will result in a narrative summary of individual study findings.

29. * Analysis of subgroups or subsets.

State any planned investigation of ‘subgroups’. Be clear and specific about which type of study or participant will be included in each group or covariate investigated. State the planned analytic approach.

If possible (based on reported data in the included studies), planned subgroup analyses will be performed to assess the effects of population and CWI characteristics (type, temperature, and duration).

30. * Type and method of review.

Select the type of review, review method and health area from the lists below.

Type of review

Cost effectiveness

No

Diagnostic

No

Epidemiologic

No

Individual patient data (IPD) meta-analysis

No

Intervention

No

Living systematic review

No

Meta-analysis

Yes

Methodology

No

Narrative synthesis

No

Network meta-analysis

No

Pre-clinical

No

Prevention

No

Prognostic

No

Prospective meta-analysis (PMA)

No

Review of reviews

No

Service delivery

No

Synthesis of qualitative studies

No

Systematic review

Yes

Other

No

Health area of the review

Alcohol/substance misuse/abuse

No

Blood and immune system

No

Cancer

No

Cardiovascular

No

Care of the elderly

No

Child health

No

Complementary therapies

Yes

COVID-19

No

Crime and justice

No

Dental

No

Digestive system

No

Ear, nose and throat

No

Education

No

Endocrine and metabolic disorders

No

Eye disorders

No

General interest

No

Genetics

No

Health inequalities/health equity

No

Infections and infestations

No

International development

No

Mental health and behavioural conditions

No

Musculoskeletal

No

Neurological

No

Nursing

No

Obstetrics and gynaecology

No

Oral health

No

Palliative care

No

Perioperative care

No

Physiotherapy

No

Pregnancy and childbirth

No

Public health (including social determinants of health)

No

Rehabilitation

No

Respiratory disorders

No

Service delivery

No

Skin disorders

No

Social care

No

Surgery

No

Tropical Medicine

No

Urological

No

Wounds, injuries and accidents

No

Violence and abuse

No

31. Language.

Select each language individually to add it to the list below, use the bin icon to remove any added in error.
English

There is an English language summary.

32. * Country.

Select the country in which the review is being carried out. For multi-national collaborations select all the countries involved.

Australia

33. Other registration details.

Name any other organisation where the systematic review title or protocol is registered (e.g. Campbell, or The Joanna Briggs Institute) together with any unique identification number assigned by them. If extracted data will be stored and made available through a repository such as the Systematic Review Data Repository (SRDR), details and a link should be included here. If none, leave blank.

34. Reference and/or URL for published protocol.

If the protocol for this review is published provide details (authors, title and journal details, preferably in Vancouver format)

Add web link to the published protocol.

Or, upload your published protocol here in pdf format. Note that the upload will be publicly accessible.

No I do not make this file publicly available until the review is complete

Please note that the information required in the PROSPERO registration form must be completed in full even if access to a protocol is given.

35. Dissemination plans.

Do you intend to publish the review on completion?

Yes

Give brief details of plans for communicating review findings.?

The review will be submitted to a relevant journal.

36. Keywords.

Give words or phrases that best describe the review. Separate keywords with a semicolon or new line. Keywords help PROSPERO users find your review (keywords do not appear in the public record but are included in searches). Be as specific and precise as possible. Avoid acronyms and abbreviations unless these are in wide use.

Systematic review; meta-analysis; cold water immersion; ice bath; adults; physiological; psychological;

cognitive; effect

37. Details of any existing review of the same topic by the same authors.

If you are registering an update of an existing review give details of the earlier versions and include a full bibliographic reference, if available.

None

38. * Current review status. [1 change]

Update review status when the review is completed and when it is published.New registrations must be ongoing so this field is not editable for initial submission.

Please provide anticipated publication date

Review_Completed_not_published

39. Any additional information.

Provide any other information relevant to the registration of this review.

40. Details of final report/publication(s) or preprints if available.

Leave empty until publication details are available OR you have a link to a preprint (NOTE: this field is not editable for initial submission). List authors, title and journal details preferably in Vancouver format.

Give the link to the published review or preprint.
