# Supplementary material for: Effects of cold-water immersion on health and wellbeing: A systematic review and meta-analysis
Source: PLoS One. 2025 Jan 29;20(1):e0317615. doi: 10.1371/journal.pone.0317615 (PMC11778651; doi:10.1371/journal.pone.0317615)
Supplement: S9 File — (PDF) [file pone.0317615.s009.pdf]

	Inclusion	Exclusion
**Population**	• Healthy adults ≥18yrs	• Tier 3 and above Athletes (McKay et al., 2022)<br>• Studies evaluating effects of CWI on any population involving chronic illness or musculoskeletal injury
**Intervention**	• Studies that involve acute or long-term exposure to CWI (e.g., 1 session or 12 weeks of regular sessions)<br>• CWI exposure must be conducted through cold shower, ice bath or cold plunge<br>• Water temperature must not exceed 15˚C<br>• CWI can be any time of day including pre or post exercise<br>• Minimum CWI exposure time of 30 seconds<br>• Immersion at/above chest level	CWI exposure through:<br>• Cryotherapy<br>• Accidental exposure<br>• Single limb exposure<br>• Wearing protective gear (wet suit etc)
**Comparator**	• Any comparator	
**Outcome(s)**	Physiological health and wellbeing response outcomes after exposure related to:<br>• Sleep<br>• Stress<br>• Fatigue<br>• Energy<br>• Skin health<br>• Immunity<br>• Inflammation markers<br>Psychological health and wellbeing response outcomes after exposure related to:<br>• Mental wellbeing<br>• Depression<br>• Anxiety<br>• Stress<br>• Mood<br>• Cognitive function<br>• Concentration<br>• Alertness<br>• Focus	Response outcomes related to:<br>• Muscle soreness<br>• Muscle function<br>• Sport performance<br>• Athlete recovery<br>• HR variability<br>• Pain

**Study types**	Randomised control trials, published in peer-reviewed sources (full length conference reports will be included).	• Observational studies<br>• Quasi-experimental studies<br>• Reviews<br>• Expert opinions<br>• Commentaries<br>• Letters to editor<br>• Studies on animals<br>• Conference abstracts
