# Supplementary figures and images for: Effects of cold-water immersion on health and wellbeing: A systematic review and meta-analysis
Source: PLoS One. 2025 Jan 29;20(1):e0317615. doi: 10.1371/journal.pone.0317615 (PMC11778651; doi:10.1371/journal.pone.0317615)

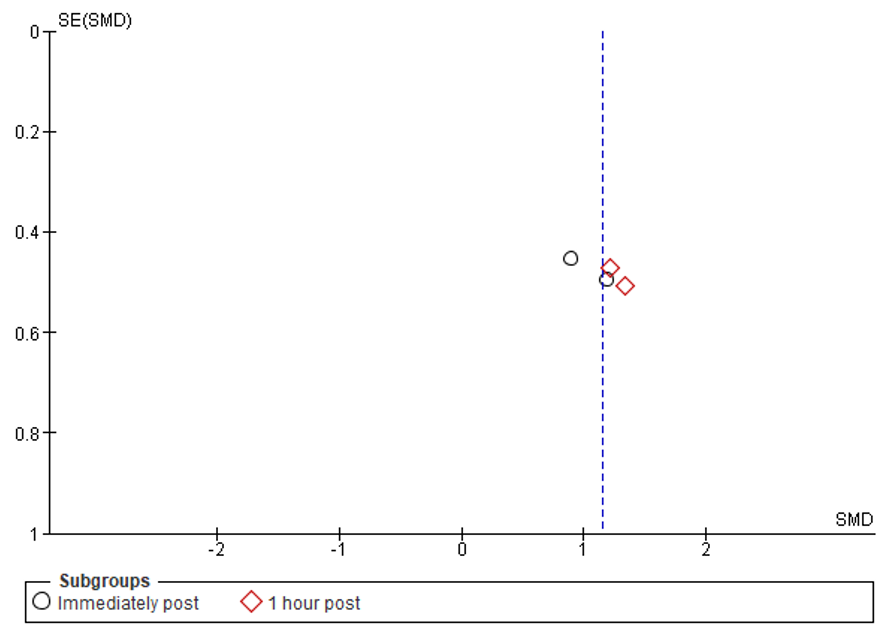

Supplement: S10 Fig — (TIF) [file pone.0317615.s010.tif]

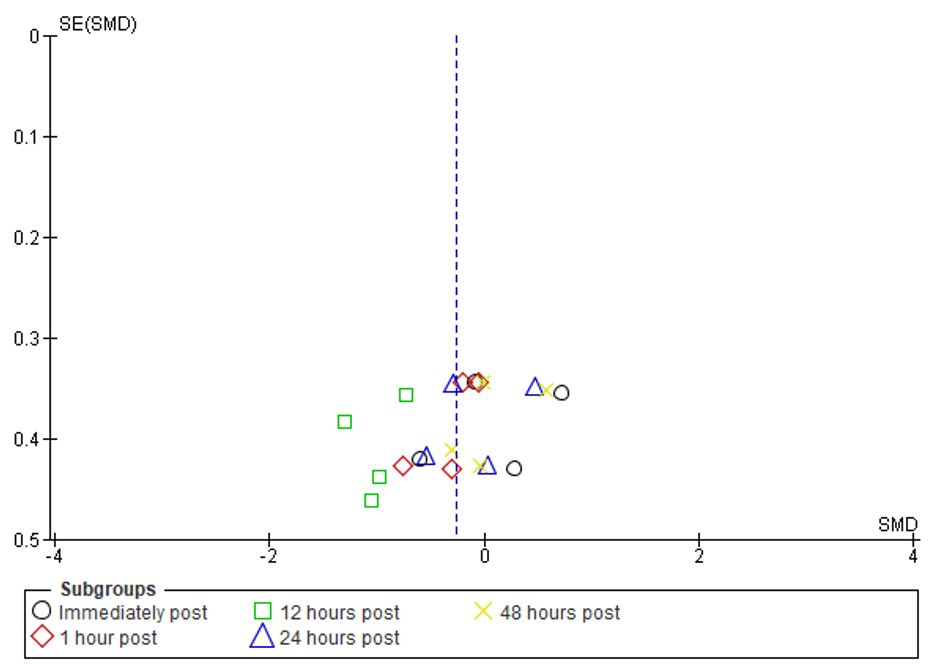

Supplement: S11 Fig — (TIF) [file pone.0317615.s011.tif]

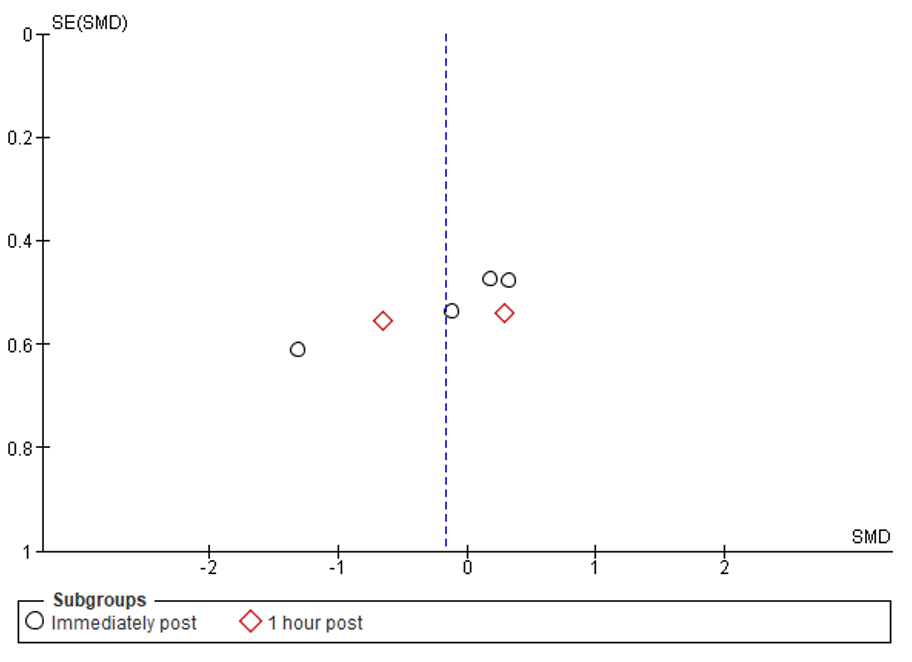

Supplement: S12 Fig — (TIF) [file pone.0317615.s012.tif]

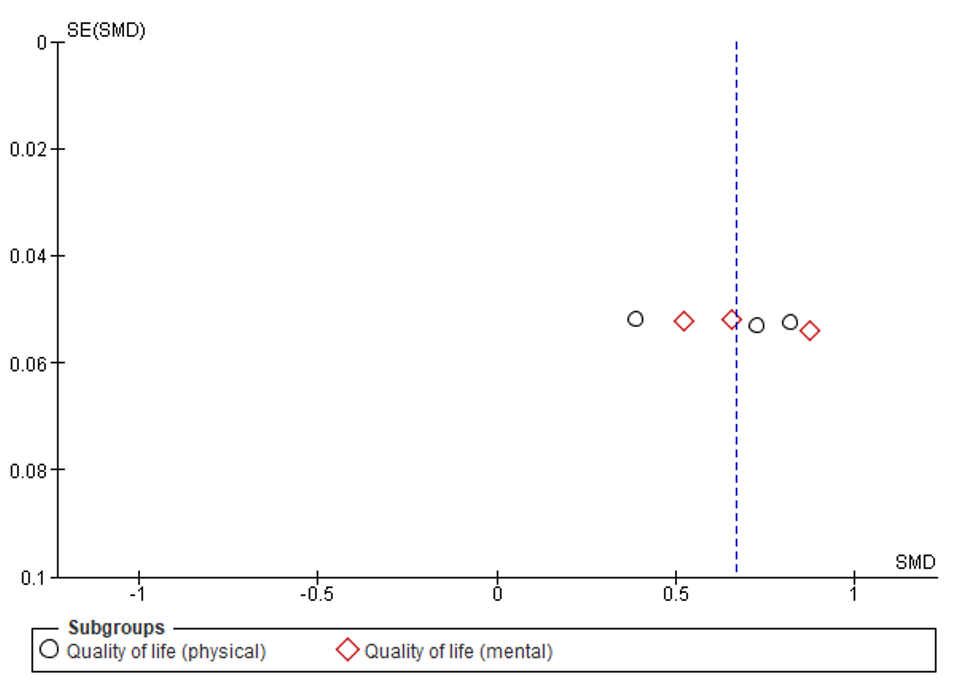

Supplement: S13 Fig — (TIF) [file pone.0317615.s013.tif]
